# Qualitative Classification of Lubricating Oil Wear Particle Morphology Based on Coaxial Capacitive Sensing Network and SVM

**DOI:** 10.3390/s22176653

**Published:** 2022-09-02

**Authors:** Ling Zhu, Xiangwen Xiao, Diheng Wu, Yishou Wang, Xinlin Qing, Wendong Xue

**Affiliations:** School of Aerospace Engineering, Xiamen University, Xiamen 361005, China

**Keywords:** wear particles, wear particle morphology, qualitative classification, support vector machine, parameter selection, coaxial capacitive sensing network

## Abstract

In addition to lubricating and cooling, aero-engine lubricating oil is also a transport medium for wear particles generated by mechanical wear. Online identification of the number and shape of wear particles is an important means to directly determine the wear state of rotating parts, but most of the existing research focuses on the identification and counting of wear particles. In this paper, a qualitative classification method of wear particle morphology based on support vector machine is proposed by using the wear particle capacitance signal obtained by the coaxial capacitive sensing network. Firstly, the coaxial capacitive sensing network simulation model is used to obtain the capacitance signals of different shapes of wear particles entering the detection space of different electrode plates. In addition, a variety of intelligent optimization algorithms are used to optimize the relevant parameters of the support vector machine (SVM) model in order to improve the classification accuracy. By using the processed data and optimized parameters, a SVM-based qualitative classification model for wear particles is established. Finally, the validity of the classification model is verified by real wear particles of different sizes. The simulation and experimental results show that the qualitative classification of different wear particle morphologies can be achieved by using the coaxial capacitive sensing network signal and the SVM model.

## 1. Introduction

**Figure 1:**
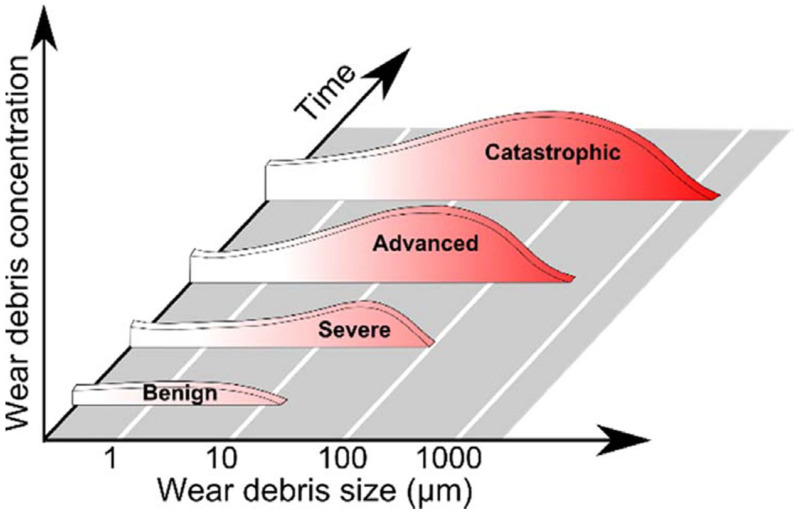
The relationship between the size and concentration of wear particles and the wear state of rotating parts.

Lubricating oil system is an important part of an aero-engine. In addition to lubricating and cooling, lubricating oil is also a transport medium for wear particles generated by the wear of mechanical rotating parts. As the engine operates normally, the lubricating oil contains a constant amount of wear particles and the size of the particles is small. When abnormal wear occurs, the concentration and size of wear particles will increase, and the morphology of wear particles will be more abundant and diverse. Therefore, if the information related to the lubricating oil wear particles (such as the number of wear particles, the size of wear particles and the shape of wear particles) can be obtained accurately and comprehensively in the first time, it can not only determine the health status of the lubricating oil system, but also provide important data support for evaluating the wear condition of mechanical rotating parts, as well as provide a basis for determining the maintenance requirements of key engine components [1]. When the rotating parts are in good condition, the size of the wear particles produced is between 1 μm and 10 μm; when the parts are abnormal, the size of the wear particles is between 20 μm and 100 μm; once the size of the wear particles exceeds 100 μm, the parts are in danger of failure. The relationship between the size and concentration of wear particles and the wear state of rotating parts is shown in Figure 1.

At present, the detection of lubricant wear particles mainly includes offline and online methods [2]. Offline methods mainly include ferrography [3] and spectroscopy [4], where lubricating oil samples are collected periodically and sent to the laboratory for testing. Researchers have developed a variety of new methods and technologies for offline detection, including ferrography image processing of wear particles based on neural network, automatic classification of wear particles, etc. [5,6,7,8]. Although these offline methods can provide more comprehensive information on oil wear particles (such as the material properties of wear particles and the three-dimensional morphology of wear particles), they are time-consuming, require many instruments, and are complex and expensive, and cannot achieve real-time acquisition and timely feedback of wear particle information.

On-line monitoring of aero-engine lubricating oil wear particles can monitor the internal state of the engine in a timely manner, and predict engine failures by analyzing the monitoring data, which improves the safety and reliability of aircraft operation. Through the online technology of lubricating oil wear particles, the crew can find faults in time and make corresponding responses, reducing the accident rate of the aircraft; moreover, maintenance personnel can also identify the fault type and location with the help of the rich information contained in the wear particles, thereby reducing costs and improving efficiency.

At present, the online monitoring technologies of lubricating oil wear particles mainly include optical method [9], acoustic method [10] and electromagnetic method [11,12,13]. The optical method is generally closely combined with the graphic image analysis technology, and there are many types of parameters that can be detected, but it is easily affected by air bubbles and is suitable for low flow rates; the principle of the acoustic method is similar to that of the optical method; when using the wear particles, it can be monitored by acoustic energy loss, and it is impossible to distinguish between wear particles and air bubbles; the electromagnetic method can be divided into magnetic type [11,12], inductive type [14], resistance type [15], charge type [13] and capacitive type [16,17]. The magnetic type is generally suitable for monitoring ferromagnetic wear particles, while the inductive type can distinguish between ferromagnetic and non-ferromagnetic particles; the resistance type relies on the resistance change generated by the wear particles for monitoring. The size of the wear particles that can be monitored is limited; in addition, the monitoring efficiency is limited. The resistance type is easily disturbed by external factors such as temperature; friction generates energy to charge the wear particles, and the electrical charge monitors the oil wear particles by monitoring the charge of the wear particles, but cannot monitor the information such as the shape and size of the wear particles; the principle function of capacitive type is to characterize the change of the medium between the electrode plates by measuring the capacitance value of the capacitive sensor, and the change of the properties of the lubricating oil will also cause the change of the capacitance value between the electrode plates.

Most of the existing lubricating oil wear particle monitoring technologies can only identify the existence and counting of wear particles, and can not carry out qualitative classification of wear particles. When the size of wear particles in lubricating oil is greater than a certain value, the engine may be seriously worn. At the same time, the shape of wear particles can also reflect the wear type of engine rotating parts. Bowen and Anderson systematically studied the correlation between wear particle size and five wear types, as shown in Table 1 [18,19]. We can find that the morphology of wear particles has a close relationship with the size of wear particles and the cause of wear. Therefore, it is of great significance to determine the morphology of oil wear particles by means of on-line monitoring. In order to meet the accuracy requirements of on-line monitoring of lubricating oil wear particles, it is also necessary to ensure that the phenomenon of missed detection and false detection of the sensor should be minimized. In order to obtain more comprehensive information in real time and eliminate the influence of other factors on lubricating oil wear particle monitoring, the research group of the author developed a lubricating oil wear particle monitoring method based on coaxial capacitance sensor network [20].

The monitoring method of lubricating oil wear particles based on coaxial capacitance sensor network can realize the quantitative monitoring of wear particles, and reflect the size and quality of wear particles through capacitance signals. At the same time, the parallel plate electrode and the non parallel plate electrode can provide multi-dimensional information of the abrasive particle morphology. However, the qualitative classification of wear particles cannot be realized by using the signal of a single wear particle. We propose a wear particle classification model based on support vector machine. The different wear particle capacitance data measured by the different electrode plates in the sensor network are preprocessed, and they are imported into the support vector machine (SVM) model for training, and then the best parameters of the support vector machine model are obtained by using a variety of intelligent optimization methods to obtain the wear particle classification model. Then the experimental data is imported into the model for verification, and finally the qualitative identification of wear particles is realized. The innovation of the proposed method mainly includes the following three points: (1) the multi-dimensional information measured by coaxial networked sensors is used to classify the wear particles. (2) The response signals of a large number of different shapes of abrasive particles through the sensor are simulated to construct the data set required for machine learning. (3) The support vector machine algorithm is improved to more accurately classify wear particle morphology. The morphology of wear particles in lubricating oil can be monitored using our new method. This is not possible with existing methods. The proposed method can identify the morphology of wear particles. This can better reflect the wear state and wear cause of the machine.

The remaining paper is organized as follows. Section 2 introduces the qualitative classification principle of wear particle morphology based on the coaxial capacitive sensor network. Section 3 introduces the support vector machine and related optimization algorithms. Section 4 introduces the construction and verification of the qualitative classification model of wear particles. Section 5 introduces the performance verification of the qualitative classification model based on experimental wear particles. Section 6 summarizes the paper.

## 2. Qualitative Classification Principle of Wear Particle Morphology Based on Coaxial Capacitive Sensor Network

### 2.1. Working Principle of Coaxial Capacitance Sensor Network

**Figure 2:**
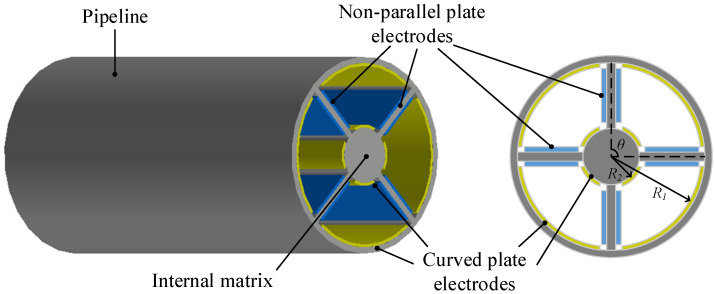
Schematic diagram of coaxial capacitance sensing network model.

At present, the existing capacitive sensors mainly have the following three problems: (1) the electrode plate spacing affects the sensitivity of the sensor, and the too small electrode plate spacing leads to the smaller oil flux in the sensor. This leads to the contradiction between sensor sensitivity and sensor efficiency; (2) the factors affecting the dielectric constant between electrode plates, such as temperature and the change of oil properties, cannot be ruled out; (3) the existing sensor electrode plate layout structure can only obtain the information of one dimension of wear particles, which is not enough to obtain the morphology of wear particles. In order to solve the above problems, the author’s research group expanded the coaxial capacitance sensor developed in [17] and created a new coaxial capacitance sensor network [20], as shown in Figure 2. The test results show that the sensor network can effectively detect wear particles of different sizes.

According to the oil pipeline structure, the area where the oil flows through the whole sensor is divided into four subspaces, and two pairs of capacitive electrodes (parallel electrode plate electrode and non-parallel electrode plate electrode) are arranged in each subspace. The interior of the sensor is divided into multiple subspaces, which solves the first problem. At the same time, because the four subspaces are not connected with each other, when there are oil wear particles in one area and no oil wear particles pass through other areas, the differential capacitance between the two areas is the capacitance caused by oil wear particles, and the influence of external factors such as temperature, flow, and oil property changes can also be filtered out, which can solve the second problem. When equipped with multiple groups of electrode plates in a subspace, one can obtain the multi-dimensional information data generated by different forms of wear particles in the movement process, which solves the third problem.

### 2.2. Qualitative Classification Method of Wear Particle Morphology

Through the coaxial capacitance sensing network, the wear particles can cause the capacitance response changes of non-parallel electrode plate electrode and parallel electrode plate electrode at the same time. A large number of different forms of wear particles produced by different wear types are placed in the coaxial capacitance sensing network. In the process of entering and leaving a detection subspace, due to the influence of flow field, the wear particles of different forms have different motion forms at different times, so that the two pairs of electrode plates in the detection subspace obtain different capacitance responses. That is, the two capacitance signals generated by two pairs of capacitor electrode plates can be used to characterize the characteristic information of wear particles passing through the detection subspace, which lays a data foundation for the qualitative classification of wear particle morphology.

**Figure 3:**
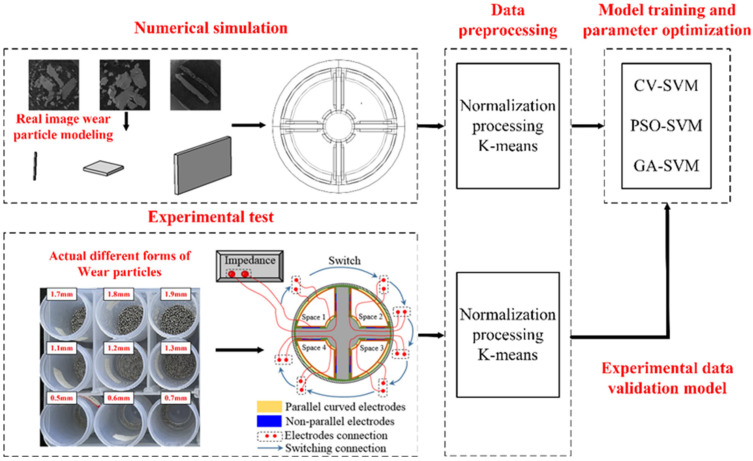
Flow chart of qualitative classification of lubricating oil wear particle morphology.

The qualitative classification of wear particle morphology essentially belongs to data classification and clustering. At present, the main classification algorithms include decision tree, support vector machine, artificial neural network, and random forest. Combining the optimization algorithm with SVM to establish a model and optimize the parameters in SVM has the advantages of fast search speed and high efficiency, which is suitable for the requirements of wear particle on-line monitoring. Firstly, two capacitance signals of different forms of wear particles passing through the detection space are obtained by numerical simulation method, normalized, and the wear particle classification model based on SVM is established. In order to improve the classification accuracy of the model, a variety of intelligent optimization algorithms are adopted to optimize the parameters of SVM to form a classification model with high accuracy. In order to classify the wear particles with different capacitance, the model is normalized to the actual size of the wear particles, and the final classification of the wear particles is carried out by using the current capacitance model. The flow chart of this method is shown in Figure 3.

## 3. Overview of Support Vector Machine and Model Optimization Algorithm

In this study, the classification model is constructed by SVM algorithm. The simulation and experimental data are preprocessed by K-means algorithm to eliminate outliers. A variety of optimization algorithms are used to optimize SVM parameters, and the algorithm with the best optimization effect is selected. The preprocessed data are imported into SVM model for training and testing, and the qualitative classification of wear particle morphology is completed. The following describes the relevant algorithms and processes used.

### 3.1. Support Vector Machine

Support vector machine [21,22] is an algorithm for data classification in machine learning. SVM is a straight line when classifying two types of linearly separable data; it is a hyperplane when classifying high-dimensional data points. The support vector is the point closest to the segmentation plane; when the distance between the data points and the hyperplane (i.e., the interval) is larger, the classifier is stronger. Support vector machine is a segmentation surface that segments the given data points, and its position changes with the support vector. By extending the one-dimensional classification line and two-dimensional classification surface in any dimension, the segmentation hyperplane can be expressed by the following formula:

(1) $$w^{Τ}x+b=0$$

where $w^{Τ}$ and b are parameters of SVM. The distance from the data point to the hyperplane is:

(2) $$d=\frac{1}{\left|\left|w\right|\right|}\left|w^{Τ}x+b\right|$$

It can be seen from (2) that at that time, the data points were located on the segmentation hyperplane; the data points lie below the split hyperplane.

**Figure 4:**
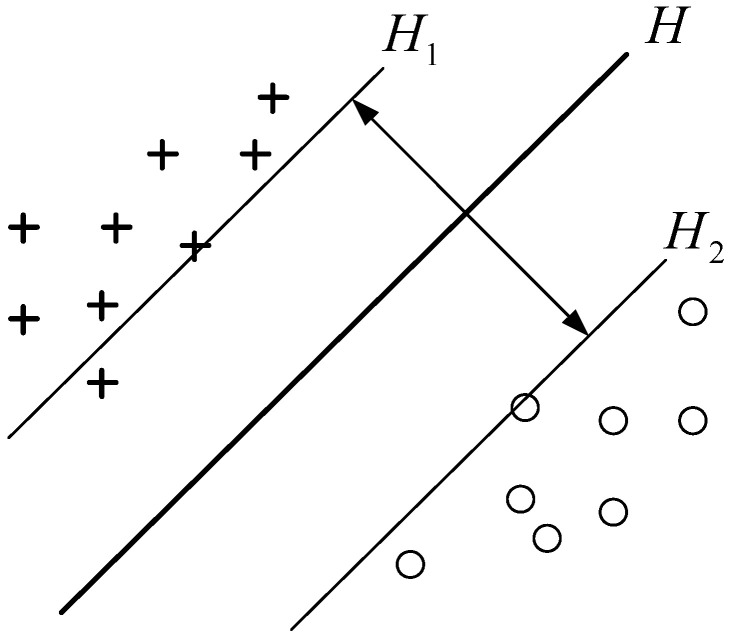
Optimal segmentation hyperplane.

After determining the interval formula, it is necessary to determine the parameters and form the hyperplane to maximize the interval between the support vector and the segmentation hyperplane. As shown in Figure 4, the hyperplane represents the optimal segmentation hyperplane and the plane representing the support vector parallel to and passing through various types of data points. Therefore, the above optimization problem is focused on maximizing the distance from hyperplane to plane and between planes, which can be directly expressed by the formula:

(3) $$\arg\underset{w,b}{\max}\left\{\underset{n}{\min}\left(y_{i}\left(w^{Τ}x+b\right)\right)\frac{1}{\left|\left|w\right|\right|}\right\}$$

where the data label is set as +1/−1.

The objective of optimization is to maximize the distance from the data point to the hyperplane. If the constraint is set to the minimum part, the optimization problem can be expressed as:

(4) $$\begin{array}{c} arg\underset{w,b}{\max}\frac{\hat{\gamma }}{\left\|w\right\|}\\ s.t\begin{array}{cc} & y_{i}(w^{Τ}x_{i} \end{array}+b)\geq \hat{\gamma },i=1,2,\cdots ,k \end{array}$$

where $\hat{\gamma }$ is the functional interval between the support vector and the segmentation hyperplane. Divide w and b by $\hat{\gamma }$, then there is $\hat{\gamma }=\min y_{i}(w^{Τ}x_{i}+b)=1$, then (3) can be simplified as,

(5) $$\begin{array}{c} arg\underset{w,b}{\max}\frac{1}{\left\|w\right\|}\\ s.t\begin{array}{cc} & y_{i}\left(w^{Τ}x_{i}+b\right)\geq 1,i=1,2,\cdots k \end{array} \end{array}$$

In order to facilitate calculation, the maximization problem is transformed into the minimum value problem:

(6) $$\begin{array}{c} arg\underset{w,b}{\min}\frac{1}{2}{\left\|w\right\|}^{2}\\ s.t\begin{array}{cc} & -y_{i}\left(w^{Τ}x_{i}+b\right)\geq 1,i=1,2,\cdots ,k \end{array} \end{array}$$

The quadratic optimization problems under the above linear inequality constraints can be solved by Lagrange multiplier method:

(7) $$L\left(w,b,\alpha \right)=\frac{1}{2}{\left\|w\right\|}^{2}-\overset{N}{\underset{i=1}{\sum }}\alpha_{i}\left[y_{i}\left(w^{Τ}x_{i}+b\right)-1\right]$$

Among them. Regard L as a function of w and b, the necessary conditions for obtaining the minimum value when the gradient is zero are:

(8) $$\theta_{D}\left(\alpha \right)=\underset{w,b}{\min}L\left(w,b,\alpha \right)$$

For w, $w=\sum_{i=1}^{N}\alpha_{i}y_{i}x_{i}$ can be obtained from $\nabla_{w}L\left(w,b,\alpha \right)=w-\sum_{i=1}^{N}\alpha_{i}y_{i}x_{i}=0$, and it can be seen that parameter w is a linear combination of input vectors; for parameter b, $\frac{\partial L}{\partial b}=-\sum_{i=1}^{N}\alpha_{i}y_{i}=0$. The dual form can be obtained by substituting the above expression:

(9) $$w^{Τ}w={\left(\underset{i=1}{\sum }N\alpha_{i}y_{i}x_{i}\right)}^{Τ}\left(\overset{N}{\underset{j=1}{\sum }}\alpha_{i}y_{i}x_{i}\right)=\overset{N}{\underset{i=1}{\sum }}\overset{N}{\underset{i=1}{\sum }}y_{i}y_{j}\alpha_{i}\alpha_{j}\langle x_{i},x_{j}\rangle$$

(10) $$W\left(\alpha \right)=\overset{N}{\underset{i=1}{\sum }}\alpha_{i}-\frac{1}{2}\overset{N}{\underset{i=1}{\sum }}\overset{N}{\underset{j=1}{\sum }}y_{i}y_{j}\alpha_{i}\alpha_{j}\langle x_{i},x_{j}\rangle$$

Then the dual problem of the original problem can be obtained:

(11) $$\begin{array}{c} \underset{\alpha }{\max}W\left(\alpha \right)\\ s.t\begin{array}{cc} & \{\begin{array}{c} \alpha_{i}\geq 0\\ \sum_{i=1}^{N}\alpha_{i}y_{i}=0 \end{array} \end{array} \end{array}$$

The optimal solution of the original problem can be approximated by the optimization of the corresponding dual problem. By solving the parameters α, then the parameters w and b are obtained, that is, the optimal partition hyperplane can be obtained.

When the data cannot be linearly distinguished, the selection of kernel function in SVM is very important. Kernel function can map the data that cannot be linearly distinguished in low-dimensional space into high-dimensional space, and this mapping can make the data that cannot be linearly distinguished in low-dimensional space separable. The definition of kernel function is:

(12) K(x,y)=〈ϕ(x),ϕ(y)〉

where, ϕ is the mapping function, and the kernel function represents the dot product of two vectors mapped in high-dimensional space. Using kernel function can effectively prevent inner product operation in high-dimensional space, which only needs to be carried out in low-dimensional space. The expressions of three commonly used kernel functions and their advantages and disadvantages are shown in Table 2. When choosing kernel function to solve practical problems, the usual methods are as follows: First, using the prior knowledge of experts to select the kernel function in advance; the second is to use the cross validation method, that is, when selecting the kernel function, try different kernel functions respectively, and summarize and select the kernel function with the smallest error. There are two very important parameters C and G in SVM model. Where G is the parameter of RBF kernel function, which represents the width of RBF kernel function; C is the parameter attached to the relaxation vector introduced by support vector machine to solve the linear inseparability of data sets. The relaxation variable can be understood as the regularization term, and C is the regularization term coefficient, which is used to balance the empirical risk and model complexity. The higher the C value, the more intolerable the error and easier to overfit. The smaller C is, the easier interference is. C is too large or too small, with poor generalization ability. G is the parameter of the RBF function selected as the kernel. This implicitly determines the data distribution after mapping to the new feature space. The larger the G, the smaller the support vector, the smaller the g value, and the more support vectors. The number of support vectors affects the speed of training and prediction. The parameters c and g have great influence on the training efficiency and accuracy of the SVM model. Subsequently, various intelligent optimization algorithms (GA, PSO, GridSearchCV) were used to optimize the combination of C and G parameters.

### 3.2. K-Means

Machine learning can be divided into supervised learning and unsupervised learning. Supervised learning can obtain a function/model from the training data set, that is, to find the relationship between features and targets. The function/model can be used to predict the final classification results, and its training set needs an input and output. There are two common supervised learning models: statistical classification and regression analysis. The most typical algorithms are K-neighbor method [23] and support vector machine. Unsupervised learning has only data, but the data has no corresponding label. The category of training data is unknown. We need to find out the rules through learning, cluster the similar data into a cluster, and minimize the gap within the class and increase the gap between the classes. Common unsupervised learning algorithms include clustering and principal component analysis [24]. K-means algorithm [25] is a basic clustering algorithm in unsupervised learning. This algorithm clusters the points in a given data set divided into classes; the specific steps are as follows:

(a) Select samples from the initialized data set as the original cluster center;
(b) The Euclidean distance from $x_{i}$ to K cluster centers of each sample is calculated respectively, and each sample is classified into the category corresponding to the cluster center with the smallest distance by using the nearest neighbor principle;
(c) For each class $c_{j}$, recalculate the cluster center $a_{j}=\frac{1}{\left|c_{i}\right|}\sum_{x\in c_{i}}x$ of the class (i.e., the centroid of all samples of the class);
(d) Repeat steps (b) and (c) until the new cluster center remains unchanged.

Given two samples $X=\left(x_{1},x_{2},\cdots ,x_{n}\right)$ and $Y=\left(y_{1},y_{2},\cdots ,y_{n}\right)$, where n is the characteristic number, the Euclidean distance between the two vectors X and Y is:

(13) $$d\left(X,Y\right)=\sqrt{\overset{n}{\underset{h=1}{\sum }}{\left(x_{k}-y_{k}\right)}^{2}}$$

### 3.3. GridSearchCV

Cross validation (CV) is a method used to determine the classification effect of the classifier. This method is to segment and combine the sample data into training data set and test data set, let the training set train the target model, judge the obtained model on the test set, and record the classification accuracy, so as to measure the performance of classifier.

**Figure 5:**
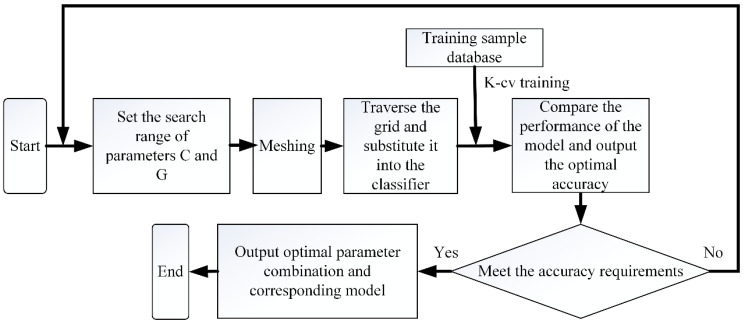
Flow chart of GridSearchCV.

The basic principle of GridSearchCV [26] is to traverse a given combination of C and G to optimize the model. Let C and G divide the grid within a certain range, use K-CV to get the corresponding classification accuracy for the given parameters, and finally get a group of parameters with the highest accuracy by searching the grid. The flow chart of GridSearchCV is shown in Figure 5.

### 3.4. Parametric Analysis of Capacitive Sensor Network 

Particle swarm optimization (PSO) is a population intelligent algorithm jointly proposed by Dr. Eberhart and Dr. Kennedy in 1995 [27]. This algorithm can simulate the predation behavior of birds [28]. Suppose there is a certain area in the natural environment where the birds are foraging, but do not know the specific location of the food source (the largest food source, i.e. the global optimal solution). In order to find the food source more effectively, the birds exchange their location information with each other in the process of exploration. Through the mutual cooperation between the groups, the whole birds will finally gather near the food source. The particle swarm optimization algorithm is inspired by this phenomenon of bird predation.

**Figure 6:**
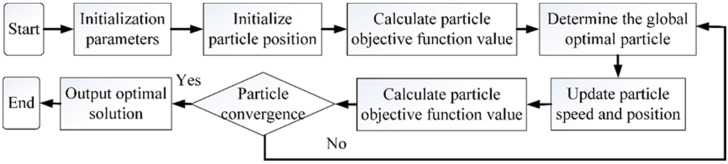
Flow chart of particle swarm optimization algorithm.

The core of particle swarm optimization algorithm is to use the information sharing and exchange of individuals in the population to make the movement of disordered population into order, so as to obtain the optimal solution of the problem. Each particle randomly assigns the corresponding initial position and initial velocity in the multi-dimensional space and then updates the position of each particle according to the particle velocity, the known global optimal position, and the known particle optimal position in order to ensure the overall optimization, and finally explore and obtain the global optimal solution. The flow chart of particle swarm optimization algorithm is shown in Figure 6. 

### 3.5. Genetic Algorithm 

**Figure 7:**
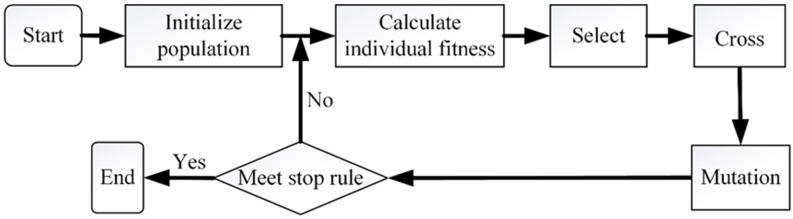
Flow chart of genetic algorithm.

Genetic algorithm (GA) conforms to the principles of “survival of the fittest” and can simulate the natural selection and genetic mechanism of biology, so as to search and obtain the corresponding optimal solution [29]. From the 1960s to the early 1970s, Professor Holland begun the preliminary research on the theory of genetic algorithm. In 1975, Professor Holland published a monograph on genetic algorithm, which became the pioneering work of genetic algorithm [30]. At present, the genetic algorithm is applied in machine learning, pattern recognition, control system, and other fields. The flow of the genetic algorithm can be represented by Figure 7.

## 4. Construction and Verification of Qualitative Classification Model of Wear Particles

### 4.1. Construction of Qualitative Classification Model Based on Simulation of Wear Particles with Different Forms

#### 4.1.1. Data Sampling and Labeling

The relationship between wear particle size and five wear types under actual working conditions is shown in Table 1. During the actual operation of the aeroengine, normal sliding wear is carried out all the time. The wear particles produced by this type of wear are generally less than 20 µm, so there is no need for monitoring. When the wear particle size is greater than 20 µm, the lubricating oil wear particles must be monitored online, and the wear particles must be classified and labeled according to the wear type, as shown in Table 3.

**Figure 8:**
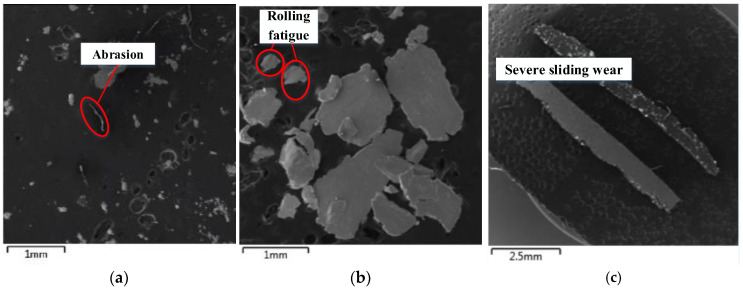
Electronic image of wear particles under actual working conditions. (**a**) Abrasion; (**b**) Rolling fatigue; (**c**) Severe sliding wear.

The electronic image of wear particles obtained from the daily engine magnetic blockage inspection report is shown in Figure 8. It can be seen that the size of wear particles is the smallest, which is extremely slender; rolling fatigue wear particles are flat, irregular and large; the wear particles of severe sliding wear are in the shape of large stripes.

**Figure 9:**
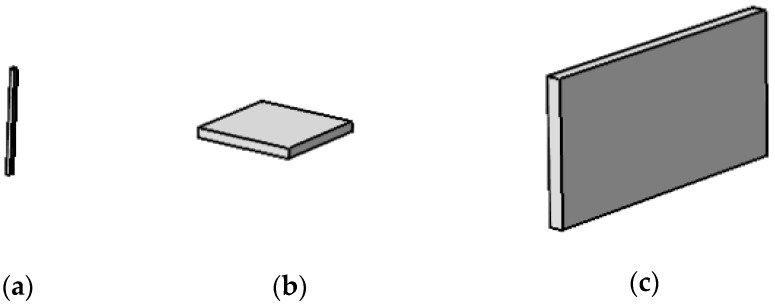
Simulation example diagram of three types of wear particles. (**a**) Abrasion; (**b**) Rolling fatigue; (**c**) Severe sliding wear.

**Figure 10:**
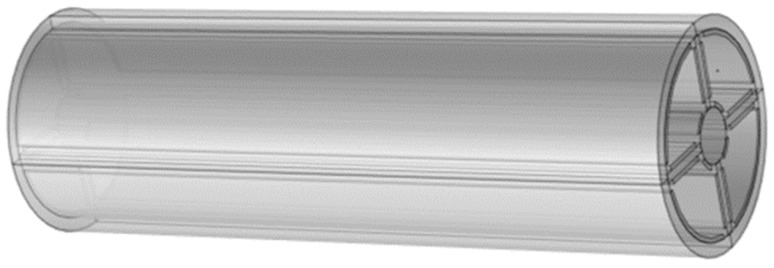
Simulation model of capacitive sensor.

According to the types and characteristics of wear particles in Table 1 and Figure 8, the model is established in COMSOL software. The example diagrams of wear particles of three wear types are shown in Figure 9. The wear particles are small and slender, the rolling fatigue particles are large flat blocks, and the severe sliding wear particles are large blocks. The equivalent diameter, thickness and slenderness ratio of each type of wear particles used for simulation vary within the corresponding range according to Table 1. In the simulation model, the cuboid model is set as the wear particle, and the material of the wear particle is iron. The specific geometric parameters of the wear particle are set as shown in Table 4. The designed coaxial sensing model is shown in Figure 10. The oil flow area in the sensor is divided into four subspaces by the internal matrix. Two pairs of different electrodes are arranged in each subspace, including non-parallel electrode plate electrodes and curved electrode plate electrodes. The oil flows through the fan space between the sensors, and the wear particles are placed in the sensor network to obtain the capacitance changes of non-parallel electrode plate electrodes and curved electrode plate electrodes. The relevant parameters of the sensor model in the finite element simulation are shown in Table 5.

Different types of wear particles are added into the sensor network, and the wear particle capacitance data of different types of electrode plates are obtained through simulation calculation. The data are normalized. The normalization formula is:

(14) $$x^{*}=\frac{x-x_{\min}}{x_{\max}-x_{\min}}$$

The normalized data is divided into training data set and test data set (60% of the training set and 40% of the test set). The training set is used to train the SVM classification model, and the test set is used to test and verify the accuracy of the trained model.

#### 4.1.2. Data Preprocessing Based on K-MEANS Algorithm

**Figure 11:**
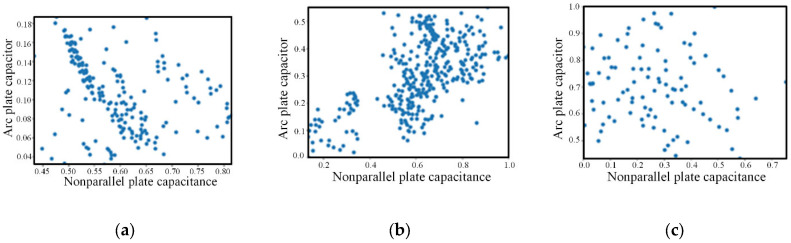
Initial data of three different types of wear capacitance. (**a**) cutting; (**b**) rolling; (**c**) sliding.

Draw each type of data separately, as shown in Figure 11. It can be seen that there are some discrete points in the data, which have a certain impact on the subsequent classification accuracy. Therefore, K-means clustering algorithm is used to eliminate the discrete points in the data. The specific steps are as follows:

(e) The data are classified according to the wear type. Each category is a separate data set, and each data set only has one cluster center;
(f) The clustering centers of different data sets are calculated based on K-means algorithm;
(g) Set the threshold to eliminate the samples far away from the cluster center.

**Figure 12:**
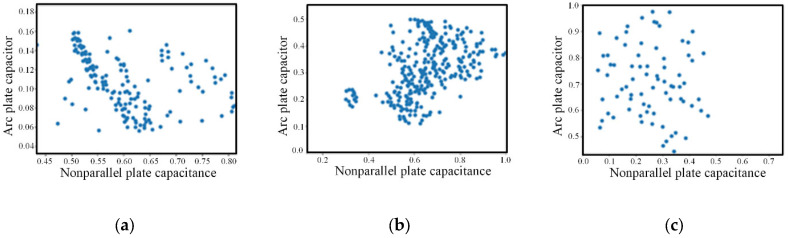
Three different types of wear capacitance data after pretreatment. (**a**) cutting; (**b**) rolling; (**c**) sliding.

The processed different kinds of data obtained through the above steps are shown in Figure 12. It can be seen that some data points far away from the cluster center were eliminated.

#### 4.1.3. Selection of Classification Model Parameters by Support Vector Machine

The kernel function is still Gaussian radial basis function (RBF) in the qualitative classification model of wear particles under actual working conditions. Then the parameters to be further selected in SVM model are mainly penalty parameter C and kernel parameter G. Therefore, grid search method, particle swarm optimization algorithm and genetic algorithm are used to determine parameter C and G respectively. We use three algorithms to determine three groups of C and G, and then select the group of C and G with the highest accuracy of the classification model. The parameters determined by the three methods and the accuracy of the classification model using the parameters are shown in Table 6.

**Figure 13:**
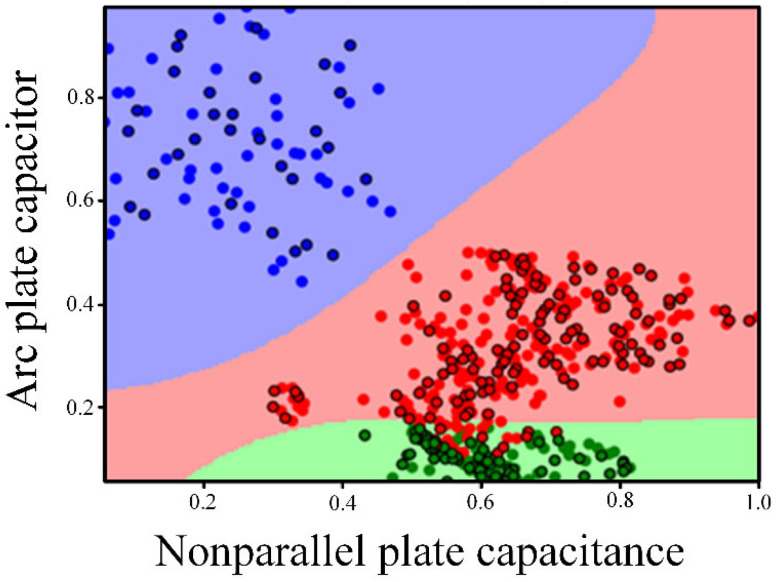
Qualitative classification results of wear particles under actual working conditions.

It can be seen from Table 6 that when the optimal parameters obtained by granular genetic algorithm (GA) are used as the parameters of the model, the accuracy of the training set and the accuracy of the test set of the final trained model are relatively high, so the penalty parameter C = 7.65 and the kernel parameter G = 7.67 are selected. The final model classification diagram is shown in Figure 13, in which blue represents sliding type, red represents rolling type and green represents cutting type. It can be seen from Figure 13 that three different types of wear particles can achieve good classification, and the accuracy verified by the test data is 96.3%, indicating that the model has good classification effect.

## 5. Performance Verification of Qualitative Classification Model Based on Experimental Wear Particles

### 5.1. Experiment Setup

**Figure 14:**
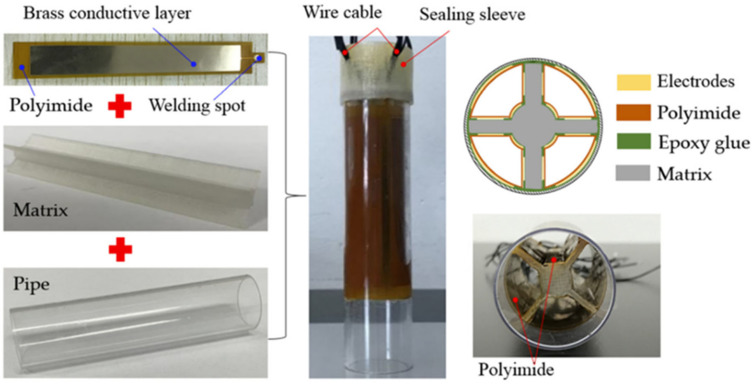
Schematic diagram of sensor.

**Figure 15:**
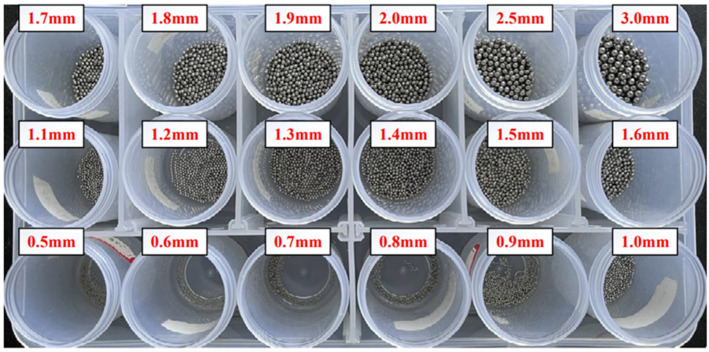
Wear particles used in the experiment.

The experiment aims to verify the classification model. The length of shaft capacitance sensor network is 80 mm, the outer radius of sensor network is 11 mm, the inner core radius of matrix is 3 mm, and the thickness of pipe is 1.5 mm. The material of the electrode plate is a flexible film. PI (polyimide) is used to encapsulate copper into a flexible film. The material used for the matrix is PLA, which is obtained by 3D printing. The object is shown in Figure 14. Metal balls are added to the sensor network to obtain response data. Metal particles with a diameter of 0.5~3.0 mm are used, as shown in Figure 15.

### 5.2. Dividing Data and Labels

The size of the wear particles used in the experiment is 0.5~3 mm. The wear particles with the same size are simulated in COMSOL multiphysics finite element analysis software, so as to obtain the wear capacitance data of different types of electrode plates in the sensor network. Classify and label the simulation data, as shown in Table 7. Then normalize the simulated wear capacitance data according to Formula (14) and divide the normalized data, in which 60% of the data is used as the training set to train the SVM classification model, and 40% of the data is used as the test set to test and verify the accuracy of the trained model.

### 5.3. Selection of Classification Model Parameters Based on Support Vector Machine

Common kernel functions can be seen from Table 2. Gaussian radial basis function (RBF) is selected in this paper. Grid search method, particle swarm optimization algorithm and genetic algorithm are used to select the optimal parameters. The parameters determined by the three methods and the accuracy of the classification model using the parameters are shown in Table 8.

**Figure 16:**
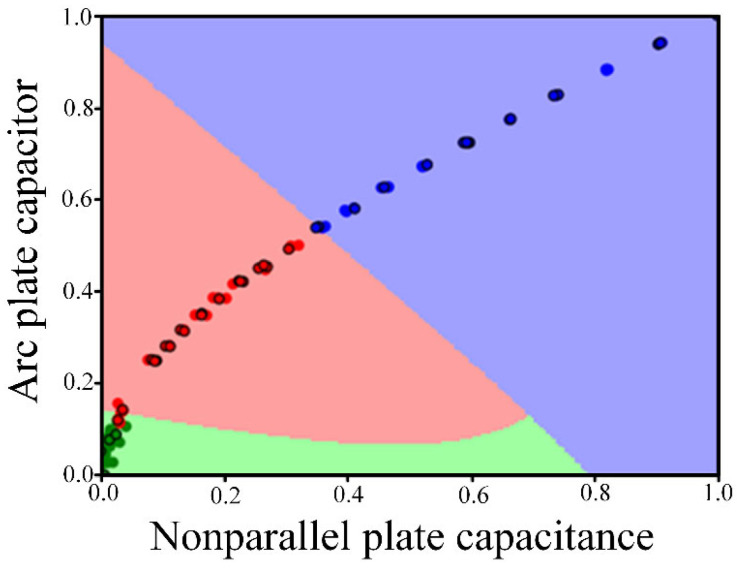
Qualitative classification results of wear particles used in the experiment.

It can be seen from Table 8 that when the optimal parameters obtained by particle swarm optimization (PSO) are used as the parameters of the model, the accuracy of the training set and the accuracy of the test set of the final trained model are relatively high. Therefore, the penalty parameter C = 8.45 and the kernel parameter G = 4.54 are selected. The final model classification diagram is shown in Figure 16, in which blue represents big type, red represents medium type and green represents small type.

Normalize the data actually measured in the experiment according to Formula (14), and then substitute it into the above classification model to compare whether the original type of data is consistent with the type obtained by the classification model. By substituting 72 groups of data into the classification model, it is concluded that the classification results of 58 groups of data are the same as the original type of data, that is, the final classification accuracy is 80.56%, and the classification effect is good. The above results show that the qualitative classification model of wear particles trained by this method has good classification effect, so it can be extended from the qualitative classification model of wear particles used in the experiment to the qualitative classification model of wear particles under actual working conditions.

## 6. Conclusions

This study proposed a classification method for wear particle morphology by using support vector machine and coaxial capacitive sensing network. The coaxial networked capacitance sensors composed of parallel plate electrodes and non-parallel plate electrodes can provide multi-dimensional information of wear particles. The data coming from simulation and experiment based on coaxial sensors are respectively used to build the training model and test model of SVM. The SVM parameters are optimized by a variety of intelligent optimization algorithms to improve the classification accuracy of the model. The comparison between our proposed method and the most advanced lubricating oil wear particle monitoring sensor is shown in Table 9. 

Through the numerical model and experimental verification, the following conclusions can be drawn:

1. The designed multi-group electrode plates can effectively monitor the wear particles on a micro scale. Coaxial capacitive sensor network can obtain multi-dimensional information characterizing wear particles.
2. The optimized SVM can accurately classify the shape of lubricating oil wear particles. The classification accuracy of simulation data is 96.35%, and that of experimental data is 95.24%.
3. The coaxial capacitance sensor network and support vector machine can be used to qualitatively classify the morphology of lubricating oil wear particles.

It can be seen that compared with the most advanced methods, the method proposed by us can more comprehensively and accurately monitor the wear particles in the lubricating oil.

## Figures and Tables

**Table 1:** Characteristics of wear particles produced by five types of wear.

Wear Type	Wear Characteristics			
	Equivalent Diameter (µm)	Thickness (µm)	Slenderness Ratio	Form
Rubbing	0.5–15	0.15–1	3:1–10:1	Minimal shape
Cutting	25–100 (length)	2–5 (width)	12:1–20:1	Slender type
Rolling contact fatigue	10–100	1–10	10:1	Block/flat
Sliding and rolling fatigue	NA	NA	4:1–10:1	Irregular shape
Severe sliding wear	>15	NA	10:1	Striped

**Table 2:** Three common kernel functions.

Kernel Function	Expression	Advantage	Shortcoming
Linear	K(x,y)=x∗y	Simple and strong interpretability.	It can only solve the linear separable problem.
RBF	$K\left(x,y\right)=\exp\left(-\frac{{\left\|x-y\right\|}^{2}}{\delta^{2}}\right)$	It can be mapped to wireless dimension with only one parameter.	Poor interpretability, slow calculation speed, and easy over fitting.
Poly	$K\left(x,y\right)={\left(\left(x*y\right)+1\right)}^{d}$	It can solve nonlinear problems.	There are many parameters, so it is not suitable for idempotents of large order of magnitude.

**Table 3:** Classification and label of wear particles under actual working conditions.

Wear Type (Label)	Wear Characteristics		
	Equivalent Diameter (µm)	Thickness (µm)	Slenderness Ratio
cutting	25–100	2–5	12:1–20:1
rolling	10–100	1–10	10:1
sliding	>15		10:1

**Table 4:** Geometric parameter setting of simulated wear particles.

Wear Type	Geometric Parameter	Parameter Range (µm)	Parameter Step (µm)	Number of Simulation Groups
Abrasion	Width	25~100	5	256
	Depth	2~5	1	
	Height	2~5	1	
Rolling fatigue	Width	10~100	10	1000
	Depth	1~10	1	
	Height	1~10	1	
Severe sliding wear	Width	30~100	5	120
	Depth	One tenth of the width	NA	
	Height	30~100	5	

**Table 5:** Geometric parameter setting of sensor simulation model.

Name	Value	Describe
***ε*_0_**	8.854187817 × 10^−12^ F/m	Vacuum Dielectric Constant
***ε***	Variable	Dielectric relative permittivity
***l***	80 mm	Sensor network length
***R*_1_**	Parameters to be determined	Inner core radius
***R*_2_**	11 mm	Sensor network radius

**Table 6:** The optimal parameters determined by three methods and the corresponding classification accuracy.

Method	Determined Parameters	Training Set Accuracy	Test Set Accuracy
GridSearchCV	C = 1.5 G = 5.4	93.90%	94.52%
PSO	C = 7.10 G = 3.59	94.21%	95.43%
GA	C = 7.65 G = 7.67	95.43%	96.35%

**Table 7:** Classification label of wear particles used in the experiment.

Size (mm)	Type
3.0, 2.5	big
2.0, 1.9, 1.8, 1.7, 1.6, 1.5, 1.4, 1.3, 1.2, 1.1	medium
1.0, 0.9, 0.8, 0.7, 0.6, 0.5	small

**Table 8:** Parameters determined by three methods and accuracy of classification model using these parameters.

Method	Determined Parameters	Training Set Accuracy	Test Set Accuracy
GridSearchCV	C = 0.9 G = 8.70	88.71%	95.24%
PSO	C= 8.45 G = 4.55	95.16%	95.24%
GA	C= 7.24 G = 3.96	90.32%	95.24%

**Table 9:** The proposed method is compared with the most advanced monitoring sensor for lubricating oil wear particles.

Oil Wear Particle Monitoring Sensors	Wear Particle Count	Wear Particle Morphology Recognition
Capacitance Kurt counter	Realizable	Not achievable
Microchannel capacitance sensor	Realizable	Not achievable
Coaxial Capacitive Sensing Network and SVM	Realizable	Realizable

## Data Availability

The data that support the findings of this study are available from the corresponding author.
